# Comparison of the Effect of Three Abutment-implant Connections on Stress Distribution at the Internal Surface of Dental Implants: A Finite Element Analysis

**DOI:** 10.5681/joddd.2013.021

**Published:** 2013-08-30

**Authors:** Saeed Raoofi, Maryam Khademi, Reza Amid, Mahdi Kadkhodazadeh, Mohammad Reza Movahhedi

**Affiliations:** ^1^Assistant Professor, Department of Periodontics, Faculty of Dentistry, Shiraz University of Medical Sciences, Shiraz, Iran; ^2^Assistant Professor, Department of Periodontics, Faculty of Dentistry, Ahwaz University of Medical Sciences, Ahwaz, Iran; ^3^Assistant Professor, Department of Periodontics, Faculty of Dentistry, Shahid Beheshti University of Medical Sciences, Tehran, Iran; ^4^Associate Professor, Department of Periodontics, Faculty of Dentistry, Shahid Beheshti University of Medical Sciences, Tehran, Iran; ^5^PhD, Department of Industrial Manufacturing, Sharif University of Technology, Tehran, Iran

**Keywords:** Biomechanics, dental implant/abutment, finite element, stress

## Abstract

***Background and aims.*** The aim of this study was to determine the stress patterns within an implant and the effect of different types of connections on load transfer.

***Materials and methods.*** Three different types of implant-abutment connections were selected for this study. Sample A: 1.5-mm deep internal hex corresponding to a lead-in bevel; sample B: a tri-channel internal connection; and sample C: in-ternal Morse taper with 110 degrees of tapering and 6 anti-rotational grooves. Four types of loading conditions were simu-lated in a finite element model, with the maximum von Mises stress set as output variables.

***Results.*** The maximum stress concentration at the inner surface of the fixtures was higher than the stress value in bone in all of the samples. Stress values in sample B were the lowest amongst all of the models. Any alterations in the amount and direction of the 100-N axial load resulted in an increase in fixture surfaces stress. Overall, the highest amount of stress (112 MPa) was detected in sample C at the inner surface of the fixture under a non-axial load of 300 N.

***Conclusion.*** Stress concentration decreased when the internal surface area increased. Creating three or six stops in the internal surface of the fixtures resulted in a decrease in stress.

## Introduction

Titanium is a widely accepted material in implant dentistry due to the advantages offered by its mechanical properties and excellent longevity in the jawbone.^1,2^ However, long-term follow-up studies on implants indicate that many complications occur after the prosthetic phase. These complications include soft tissue inflammation, bone loss, abutment screw loosening,^3^ abutment screw fracture,^4^ and loss of osseointegration.^5^ As is evident in recent literature, successful osseointegration occurs in more than 95% of the cases following the surgical phase, regardless of the implant system used.^6^ Therefore, it is not surprising that some authors have suggested that late failures (after more than 1 year of loading) are due to overload in 90% of cases, with 10% being attributed to periimplantitis.^7^

A thorough understanding of implant biomechanics makes it possible to optimize treatment planning and reduce any risk of functional complications and failures. The application of engineering knowledge in dentistry has contributed to an understanding of biomechanical aspects related to implantology.^8^ Finite element analysis (FEA) was initially developed in the early 1960s to solve structural problems in the aerospace industry but has since been extended to implant dentistry.^9-11^

There are a numbers of publications on the effects of implant diameters, platform switching design, ridge diameters and inclination of load applied to an implant on stress/strain patterns in the surrounding bone.^11-13^ However, information regarding stress patterns within an implant and the effects of various types of connections on load transfer are rare. A load is applied to the superstructure part of an implant, and transferred to the abutment. The abutment carries the load to the fixture and bone through the implant-abutment and implant-bone connection. This load is finally applied to the surrounding bone. Therefore, the implant-abutment connection area has an important role in modifying this load. The difference between external and internal types of abutment connection has been studied previously, although there is no information regarding biomechanical comparison of different types of internal connections. A precise and well-designed connection leads to high rotational stability. Finally, a stable interlocking fit between implant and abutment reduces the occurrence of micro-movements and guarantees that the retaining screw will remain in place without being exposed to the risk of screw loosening or screw breakage. There are different kinds of internal connections on the market, although the most reliable one has not been recognized. Therefore, this study was designed to examine the role of internal connection design on stress/strain distributions within an implant structure.

## Materials and Methods

**Figure 1:**
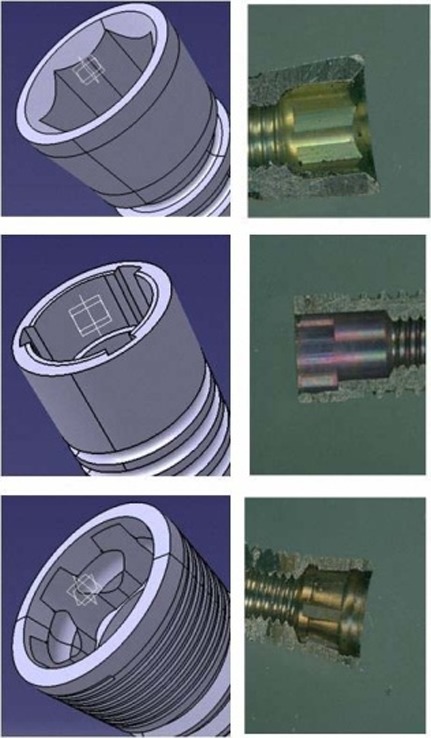
Schematic view drawn by CATIA (left) and scanned view of implant fixture (right). Top: Internal hex with lead-in bevel at occlusal part of the fixture. Middle: Tri-channel internal connection. Bottom: Internal Morse taper with 6 anti-rotational grooves.

Three different types of implant-abutment connections used in commercially well-known implant systems were selected. The features of these connections are as follows (Figure 1):

1) Sample A: 1.5-mm deep internal hex corresponding to lead-in bevel (BioHorizons Co. Birmingham, USA);

2) Sample B: tri-channel internal connection (Nobel Biocare, Goteborg, Sweden);

3) Sample C: internal Morse taper with 11 degrees of tapering and 6 anti-rotational grooves (Intra-Lock International, Inc. FL, USA).

### Model Geometry

The present study was based on real implants available on the market. Implants from different systems did not possess similar dimensions. Therefore, in order to decrease confounding factors, it was decided to model implants with nearly similar dimensions; as a result, lengths of 9.511 mm and diameters of 3.54.1 mm were selected.

In order to model the implants exactly the same as the actual form, the real implants were scanned with a high-quality scanner (Scanmaker i800, Microtech, Shanghai, China). A digital caliper (Mitutoyo Canada INC., Toronto, Ontario, Canada) that opened 20 µ was used during scanning to decrease any possible magnification errors. During the second stage, all the implants were sectioned in the vertical plane through the middle part using a wire cut machine specialized in fine cutting using electrical conductance without melting (Arunoday Machine Builders, Bangalore, Karnataka, India). The section planned was designed to create one half of an implant on one side. After this procedure, the remaining series of scans were taken from the inner surfaces of each type of implant. Therefore, it was possible to access all the data regarding shapes and dimensions of the inner and outer surfaces of implants in addition to internal connection configurations of three determined systems as real as possible. All the above data were used to produce computerized models by CATIA software (CATIA VS R18, 2008).

In order to model compact and cancellous bone, a cone beam CT image of a human mandibular bone was used. The real dimensions of cortical bone were modeled in using a computer in order to create a model of bone as close to the clinical form as possible. However, since the model of the implant may reach the lingual cortex of bone, we considered the lingual plate to be as convex as the buccal plate. According to previous studies^12^-^14^ and the CT scan image sample, the cortical bone thickness was assumed to be 2 mm. The overall dimensions of bone were 18.2 mm in height, 10 mm in mesiodistal length, and 7 mm in buccolingual width.

### Material Properties

All the materials used in the models consisted of implants, abutments, and abutment screws; compact and cancellous bones were presumed to be as homogeneous, isotropic and linearly elastic as one another. The material properties, including modulus of elasticity and Poisson’s ratio used in FE model, are listed in Table 1.

The bone-implant interface was assumed to be perfect, simulating complete osseointegration. Therefore, the connections between implant-cortical and implant-cancellous bones were designed to be bonded as well as the interface between cancellous and cortical bones. Within the implant system, FEM modeling was performed by implementing bonded conditions on the abutment-implant interfaces. The entire structure was held by setting all 6 degrees of freedom of mesiodistal surfaces of cancellous and cortical bones to zero.

**Table 1:** Physical properties of different materials used in the present study

	Modulus of elasticity	Poisson’s ratio
Cancellous bone^15^	620 Mpa	0.3
Cortical bone^16^	14000 Mpa	0.3
Abutment- abutment screw^17^	114000 Mpa	0.38
Implant^18,19^	102000 Mpa	0.35

All the models were constructed using three-dimensional 4-node tetrahedral elements. Although this type of modeling with hexahedral elements is the most compatible for linearly elastic materials, hexahedral modeling could not be used due to complex geometry. The number of elements and nodes used in this study are listed in Table 2.

**Table 2:** Number of elements and nodes in different samples

	Elements	Nodes
Sample A	44009	10950
Sample E	41723	10740
Sample C	44792	78954

### Loading Conditions

Four types of loading conditions were simulated: a) 100-N force applied vertically to the abutment surfaces; b) 100-N force directed at 15 degrees to the long axis of the implant; c) 300-N force directed axially; and d) 300-N force applied at 15 degrees direction. All the forces were applied to the entire outer surface of the abutment.

### Stress/strain Analysis

The computerized model was transferred to ANSYS software (ANSYS WB 2.0 Framework, version 12.0.1, 2009 SAS IP). All the conditions mentioned previously (material properties, interface condition, meshing and loading) were included in this software.

### FEA Data Collection

**Figure 2:**
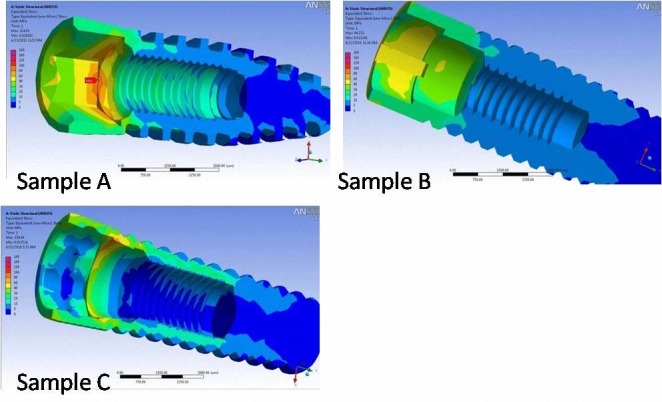
Stress distribution pattern on the buccal side of internal surface of fixture under 300 N.

**Figure 3:**
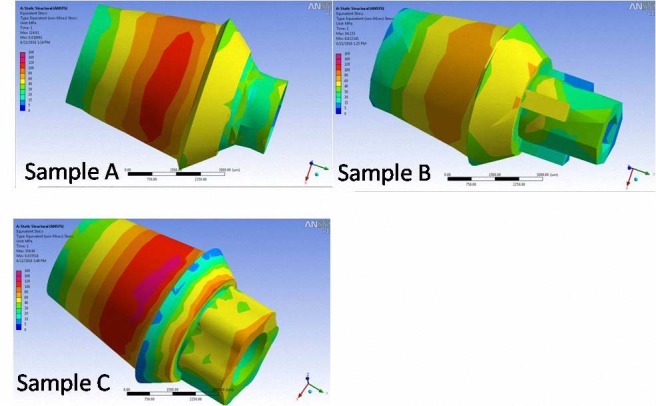
Buccal aspect of stress distribution pattern under 300-N non-axial load on the outer surface of abutment.

a) Quantitative analysis was carried out after variables were evaluated subsequent to imaginary loading at four different protocols mentioned above. These analyses were performed at the inner surface (implant-abutment interface). The maximum von Mises stresses (maximum equivalent stress) at the surface of the abutment and implant were set as output variables. Strain values were reported as micro-strain scale. The connection surfaces were divided into different areas in order to simplify the comparison (Figures 2 and 3).

b) For qualitative analysis, since the maximum concentrated stress and micro-strain values are presented as specific numbers, a comprehensive evaluation of stress should be carried out. The pattern of stress distribution was evaluated by comparing various diagrams.

## Results

### Stress Concentration at the Inner Surface of Fixtures

Generally, the maximum stress concentration at the inner surface of the fixtures was higher than the stress in bone in all the samples. Stress values in sample B were the lowest amongst all the models. However, any changes in the amount and direction of the 100-N axial load resulted in an increase in stress on fixture surfaces. Under all the four loading conditions, maximum stress values for the internal surface of the fixtures were recorded in sample C.

Overall, the highest amount of stress was recorded at the inner surface of fixtures under the non-axial load of 300 N in sample C, whereby 112-MPa stress was created.

The maximum stress concentration was detected at different positions of internal surfaces of the fixtures. Under 100-N buccolingual load, maximum stress in samples A and C were observed at apical areas, whereas in sample B, stress concentration was located at more coronal part of the connection (in particular at the tri-channel depression). Overall, sample B showed the greatest stress in the outer surface of the occlusal edge, whereas samples A and C showed maximum stress in the coronal part of connection area. Under the 300-N non-axial load, stress concentration patterns were the same as the 100-N non-axial loading (Figure 2).

### Maximum Stress at the Surface of the Abutment

The stress detected on the inner and outer surfaces of the abutment was generally higher in comparison to other sites mentioned previously. Stress values as high as 92 and 100 MPa were recorded in these areas, in particular under the 300-N load. Excluding the 100-N loads, the overall von Mises stress values were higher than 40 MPa.

With the exception of non-axial 300-N load (whereby minimum stress was observed in sample A), sample B showed the lowest value of von Mises stress concentration in comparison to the other samples. Under 100-N loads, stress concentration in sample A was almost the same as that in sample B, although this was not the case under the 300-N loading condition.

Under 100-N non-axial loading conditions, maximum stress concentration was observed in platform sections of the two samples (A and B). In sample C, maximum von Mises stress concentration was located in the apical end of the abutment. Under the same loading condition, sample A represented maximum stress values in the most coronal part of connection area in the outer surface of the abutment. In samples B and C, stress was concentrated at abutment shoulder; this was more apparent in tri-channel sites in sample B.

Under the tilted 300-N loading condition, all the samples showed maximum stress values at the platform of the inner surface of the abutment. At the outer surface, maximum stress concentration was located at the abutment shoulder of samples A and B. Sample B showed the secondary peak of stress at the tri-channel area. In sample C, maximum von Mises stress value was observed in the most coronal part (Figure 3).

**Table 3:** Stress concentration on the surfaces of fixtures and abutments

Stress (MPa) Area	Sample A 100 N	300 N	100 N tilt	300 N tilt	Sample B 100 N	300N	100 N tilt	300 N tilt	Sample C 100 N	300 N	100 N tilt	300 N tilt
Fixture / Internal surface	22.5	65	35	60	9.5	28	20	35	25	82	40	112
Abutment / Internal surface	24	65	30	92	15	50	21	65	25	65	30	100
Abutment / External	15	50	20	59	15	39	18	67	20	60	30	100

**Table 4:** Results of some other studies about stress distribution within implant systems

Study	Loading	Max stress internal (MPa)	Stress concentration
Van Staden,^26^ 2008	200-500-1000 vertical	Internal hex model: 273-820 External hex model: 349-1047	Internal hex model: At coronal part of screw and abutment interface a External hex model: the interface between inferior surface of screw and crown
Van Staden,^20^2008	200,500,1000: 45 oblique	50-250 Mpa	Internal and external first thread of implant
Rodrigues,^15^ 2009	1oo-axial 50-BL	100 N: 26.4 for abutment	In Abutment: At the point where the threads ended on the abutment’s internal screw, accentuating the threads’ sharp edges.
Qian,^27^ 2009	0-85 degree BL 200 N	Vertical load: 35-85 Oblique load: 40-120	In the thread tip of the implant in the neck region near the cortical bone and in the apical region close to the cancellous bone.
Assuncao,^12^ 2009	133N Oblique load : 30 degree	Fixture with thread: - Implant:1.455 - Screw:803.7 - Crown:1.151 Fixture without thread: - Implant:681.53 - Screw:791.58 - Crown:1187	Around implant neck In the fixture and some parts of abutment next to neck of implant. Also in coronal area of screw- abutment interface
Kong,^24^ 2009	100 and 30 N force in Vertical and oblique directions.	AX load: 3 mm: 10 BL load: 3mm:18.53	In Implant neck and in those part of abutment which connected to neck of implant

## Discussion

Stress concentration around abutment structures, implants and teeth has been evaluated to prevent failures of the prosthetic structures and implants. The majority of previous FEA studies have not modeled real implants. In our modeling, resemblance to real models was achieved as much as possible. Therefore, the results of this study may allow a more logical selection of appropriate internal connection systems. In addition, our results may be useful in designing new implant systems with reduced biomechanical risk factors, leading to lower rates of failure.

Distribution and magnitude of stresses within an implant are influenced by the implant dimensions and geometry as documented by some authors.^20-22^ Catastrophic mechanical failure of an implant may occur by implant fatigue,^21^ implant fractures, veneering resin/ceramic fractures or other mechanical retention failures.^20,22^ Therefore, from both engineering and clinical perspectives, an important criterion in designing an implant is to include a geometry that can minimize mechanical failures caused by an extensive range of loading.

The internal connection between different parts of the implant systems has a significant role in transferring masticatory load from the occlusal plane to the fixture and surrounding bone. Understanding the biomechanical aspects of different types of connections, (in particular the abutment implant connection), and their effects on stress/strain fields in implant/bone systems may assist in reducing the risk of implant failure.

Successful modeling depends on accurate stimulation of geometry and surface structure of the implant, material characteristics of the implant and jawbone, loading support conditions, and the biomechanical implant-jawbone interface.^23^ In this study, scanned images of the actual commercially available implants were used.

### Boundary Condition of Modeling

**Figure 4:**
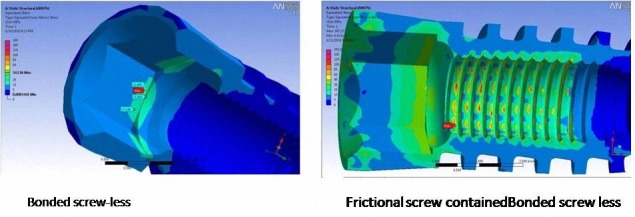
Stress distribution pattern on the internal surface of abutment, in two variations in sample A used in the pilot study.

One of the most important criteria in FEA is the characteristic features of connection between various components. Among ‘fixed bond’, ‘slip contact’ (frictional) and ‘non-linear contact’ interface boundary conditions, numerous finite element models have shown remarkable differences in the above values, and occasionally in stress distributions.^24^ Experimental evidence remains too limited for choosing the most realistic interface boundary condition. Removal of implants with rough surfaces frequently results in fractures within the bone far from the implant surface,^19^ suggesting the possible existence of an implant-bone ‘bond’. This study simulated an osseointegrated implant with a screwed rough surface; therefore, a ‘fixed bond’ condition was set as an approximation at its interface with bone. It was not possible to reach a conclusion regarding the most appropriate option for the condition of the implant-abutment interface. High stress concentration values up to 238 MPa in the screw-containing model indicated that this kind of modeling was not a suitable option for further analysis (Figure 4).

According to previous literatures, the yield strength of titanium alloy and pure titanium is in the range of 16055025 and 24240 MPa, respectively. Therefore, the mentioned results regarding stress values may be considered relatively unrealistic. The high stress levels detected in the screw may be used as an explanation for the relatively high rate of screw loosening as a well known clinical complication. However, a screw used in a mechanical structure is usually considered as a device for intimate contact between various components rather than a key element for stress shielding/distribution. Adding a screw structure to our models increased the element models and meshing complexity. Such complex models were not concomitant with increasing the reliability of the results, as all of the various types of modeling showed almost similar values for stress concentration.

The lowest stress values in fixture and abutment surfaces were detected in sample B, and the data were obtained under axial and non-axial loads of 100 N, and axial load of 300 N. However, under the non-axial 300-N load, the von Mises stress value was higher than in sample A. It was concluded that the 3-channel design of abutment connection used in sample B may be useful under normal forces, although the reaction would be different under magnified loads. In general, the highest stress values were observed under the non-axial 300-N load. It was possible to detect stress values above 100 MPa in this situation. Fortunately, the majority of stress-related fractures occurred in the titanium-made structure of the abutment and fixture. Therefore, stress concentrations at bone level showed a significant decrease in comparison to internal surface stress. It is apparent that the materials used in the above components require a standard quality to prevent fatigue fracture.

Sample C represented the highest stress values, especially under axial and non axial 300-N loads, which can be explained by restricted areas of intimate connection between the outer surface of the abutment and inner surface of the fixture. Therefore, it may be concluded that the stress was concentrated in a relatively small area. It is not surprising that the highest stress value detected in our study belonged to the internal surface of the fixture in sample C (112 MPa under non-axial 300-N load). Stress values increased up to two times under the 15-degree 100-N load, and more than three times by increasing the load from 100 N to 300 N. It appears that the components of the system may be more susceptible to the magnitude of load rather than the angle of force, although it should be mentioned that a small angle load (15 degrees) was analyzed in the present study. Higher angled forces should be evaluated in the same FEA models. Although we could not find any similar data in previous studies, Table 4 shows the results of other studies regarding stress distribution within implant systems.

Various kinds of FE modeling must be evaluated in further studies in order to select the most appropriate model. Selecting the friction versus bonded models may be a challenging issue in future studies. There were certain limitations in this study, some of which are universal in all FEAs, and others which can be managed more appropriately. It should be noted that several assumptions have been made in the present simulations: 1) the interface between the screw like implant and cortical/cancellous bones were completely bonded although this may not be the case in clinical conditions; 2) the two bone layers were assumed to be linearly elastic, whereas a nonlinear assumption may be more appropriate for the simulation of the jawbone, (given that basic material data were available). Certain components such as crowns in the implant assembly may cause different effects on stress/strain fields, although the crown was not included in the models in the present study. Therefore, the present models cannot provide absolute and realistic values of stress and strain in the jawbone/implant system of an actual model, and thus may not be quantitatively validated by a clinical study. However, for a comparative study, such simplifications are considered to be reasonable as far as the constructed models can reflect the clinical situation; 3) The aim of this study was not to replicate exact in vivo stresses but rather to illustrate possible differences in stress distribution of commercially available implant systems. Therefore, further analysis to evaluate the effect of different diameters and lengths of fixtures and abutments in each kind of connection may be necessary.

## Conclusion

The stress concentration and distribution patterns in internal connections between abutments and fixtures were not similar to the values obtained in bone. Stress analysis in abutment-fixture connections clearly showed that an increase in the surface area of the components may lead to a reduction in stress concentration. Creating three or six stops in the internal surface of the fixture resulted in a decrease in stress. Internal connections in 360 degrees without any limitation in circulation caused certain uneven stress concentrations.

## Acknowledgments

This study was supported by Vice Chancellor for Research Affairs, Shiraz University of Medical Sciences. The authors thank the following Iranian companies for supplying the fixtures used in this research: Fanavari Pishrafte Zarrin Co., Hengam Dandan Co., and Almas Rooyan Pars Co., which are distributors of BioHorizons, Nobel Biocare, and Intra Lock system, respectively.
